# Supplementary material for: A structured multi-head attention prediction method based on heterogeneous financial data
Source: PeerJ Comput Sci. 2023 Nov 17;9:e1653. doi: 10.7717/peerj-cs.1653 (PMC10703059; doi:10.7717/peerj-cs.1653)
Supplement: Supplemental Information 1 [file peerj-cs-09-1653-s001.zip › data/data description.docx]

This paper includes three types of data: investor comments, technical indicators, and financial indicators.

Investor comments: The data is sourced from investor comments in the stock forum of East Money. The data includes stock codes, user views, investor comments, and posting time. The stock code represents the source of the data from the forum, and the view count reflects the number of clicks and views received by each post from other investors. The investor comments represent the titles of each post, while the posting time indicates when the post was published. Data processing: After data cleaning to remove invalid data (such as symbols, numbers, emoticons, etc.), the text is segmented into Chinese characters, punctuation marks, and encoded using a text tokenizer. The segmented elements (tokens) are then converted into tensors suitable for training and become input vectors recognizable by the model.

Technical indicators: Technical factor data is sourced from JoinQuant, and ten technical indicators are selected. The specific indicators and their meanings are presented in Table 3. The data is processed by handling missing values and then performing min-max normalization.

Financial indicators: Financial factor data is sourced from JoinQuant, and ten financial indicators are selected. The specific indicators and their meanings are presented in Table 4. The first four indicators in the table are daily-level data, updated daily. The remaining six indicators are quarterly data, updated every quarter, with a statistical period of one quarter. Similar to technical indicators, missing values are handled first, followed by normalization processing.
