# Supplementary material for: A structured multi-head attention prediction method based on heterogeneous financial data
Source: PeerJ Comput Sci. 2023 Nov 17;9:e1653. doi: 10.7717/peerj-cs.1653 (PMC10703059; doi:10.7717/peerj-cs.1653)
Supplement: Supplemental Information 2 [file peerj-cs-09-1653-s002.zip › 600050/code book ú¿600050ú⌐.docx]

Below is the information about the data within "600050.xls" file.:

| Columns | read | The number of views or reads for each public opinion |
| --- | --- | --- |
|  | comment | The number of comments received for each public opinion. |
|  | title | The content or title of each public opinion. |
|  | author | The nickname or username of the author who published the public opinion. |
|  | created_time | The time when the public opinion was published. |
| Data Source | investor comments in the "Stock Bar" of East Money, and the website is https://www.eastmoney.com/. | |
| Timespan | The file contains the comment data for stock 600050 from January 1st ,2020,to December 31st ,2020,on the East Money platform. | |
| Data Volume | 38595 | |

Below is the information about the data within "600050data.xls" file:

| Columns | Unnamed: 0 | the date |
| --- | --- | --- |
|  | Open | Opening price of the stock. |
|  | Close | Closing price of the stock. |
|  | High | Highest price of the stock on the given day. |
|  | Low | Lowest price of the stock on the given day. |
|  | Volume | Trading volume (number of shares traded) on the given day. |
|  | Money | The total amount traded for the SSE (Shanghai Stock Exchange) on the given day. |
|  | MACD_talib | Moving average convergence/divergence of the SSE on the given day. |
|  | pb_ratio | The price-to-book ratio, which is the stock's market price per share divided by its net asset value per share. |
|  | RSI_talib | Relative Strength Indicators of the SSE on the given day. |
|  | RSV | Raw Stochastic Value of the SSE on the t-th day. |
|  | CCI_talib | Consumer Confidence Index of the SSE on the t-th day. |
|  | pe_ratio | The price-earnings ratio, which is the stock's market price per share divided by its earnings per share. |
|  | ps_ratio | The price-to-sales ratio, which is the stock's price per share divided by its sales per share. |
|  | pcf_ratio | The price-cash flow ratio, which is the stock's market price per share divided by its cash flow per share. |
|  | inc_return | Net asset return rate (deducting non-recurring gains and losses). |
|  | roa | Return on total assets. |
|  | net_profit_margin | Net profit margin on sales. |
|  | expense_to_total_revenue | Operating expenses to total revenue ratio. |
|  | net_profit_to_total_revenue | Net profit growth rate compared to total revenue. |
|  | inc_net_profit_annual | Annual net profit growth rate. |
| Time span | The file contains the comment data for stock 600050 From January 2nd, 2019, to June 30th, 2022,on the Join Quant . | |
| Data Volume | 847 | |
